# DepActive: study protocol for a randomised controlled multicentre trial of telephone-delivered behavioural activation for the treatment of depression in older adults in primary care

**DOI:** 10.1186/s13063-024-08521-y

**Published:** 2024-10-05

**Authors:** Elin Byström, Björn Wennlöf, Inger Johansson, Lena Lönnberg, Marina Arkkukangas, Johnny Pellas, Mattias Damberg

**Affiliations:** 1 Region Vastmanland – Uppsala University Centre for Clinical Research, Vastmanland Hospital Vasteras, Västerås, Sweden; 2 Department of Public Health and Caring Sciences, Uppsala University, Uppsala, Sweden; 3 The Swedish Partnership for Mental Health in Vastmanland, NSPH, Västerås, Sweden; 4 Centre for Clinical Research Sörmland, Uppsala University, Eskilstuna, Sweden; 5 Department of Medicine, Sport and Fitness Science, Dalarna University, Falun, Sweden; 6 Department of Physiotherapy, School of Health, Care and Social Welfare, Malardalen University, Västerås, Sweden

**Keywords:** Behavioural activation, Depression, Depressive symptoms, Geriatrics, Older adults, Primary care

## Abstract

**Background:**

Depression is common in older adults and is related to reduced quality of life and functional ability as well as increased mortality and morbidity. Current guidelines recommend psychological treatments for the treatment of depression in adults. Studies show that about 30% of older adults with depression in Sweden receive pharmacological treatment and about 3% receive psychological treatment. However, a majority receive no treatment at all. There is a need for effective and scalable psychological treatment options for older adults with depression in primary care. Behavioural activation is an extensively evaluated, effective, and relatively simple treatment for depression that can be delivered by health care professionals without comprehensive training in psychological treatment.

**Methods:**

We will conduct a randomised controlled 2-armed parallel group multicentre trial comparing treatment as usual in primary care to a five-session telephone-delivered behavioural activation treatment as add on to treatment as usual. The current trial is open labelled. In all, 250 older adults (≥ 65 years) with depression will be recruited from primary healthcare centres in three Swedish regions. The primary outcome is depressive symptoms measured with the Montgomery Åsberg Depression Rating Scale – Self rating version (MADRS-S) after treatment and at 3- and 6-month follow-up. Secondary outcomes include depression diagnoses, activity level (self-rated and measured with accelerometer), and self-rated anxiety, daily functioning, quality of life, self-efficacy, and loneliness.

**Discussion:**

There is a need for fully powered studies of brief behavioural activation for older adults with depression delivered by telephone in a primary care context. This study has the potential to improve first-line treatment of depression in older adults in primary care, consequently reducing morbidity and mortality within this population. Increasing the availability and accessibility to effective psychological treatment for depression in older adults is needed to meet future demographic changes.

**Trial registration:**

ClinicalTrials.gov: NCT06284889. Registered February 28, 2024.

**Supplementary Information:**

The online version contains supplementary material available at 10.1186/s13063-024-08521-y.

## Background

Depression is considered one of the leading causes of the global health burden across the entire lifespan [1]. The global prevalence of clinically relevant depressive symptoms in older adults is about 30% [2], and it is about 13% for major depressive disorder [3]. A study conducted in Swedish primary care showed a depression prevalence of 15% among adults aged 60 years or older [4]. Depression in older adults is associated with reduced quality of life [5] and reduced physical, cognitive, and social functioning and increases the risk for morbidity and mortality [6]. Depressive symptoms in older adults have also been shown to be able predict rehabilitation outcomes [7]. A recent United Nations prognosis indicate a continuously growing proportion of older adults with about 25% of the population in Europe and Northern America aged 65 years or over by 2050 [8]. The high prevalence of depressive symptoms in older adults and the upcoming demographical changes stress the need for effective and scalable interventions for the treatment of depression in older adults.

However, depression is a treatable condition and there are several treatment options for depression in older adults, including antidepressant medication, psychological treatment, and physical activity [9]. In the guidelines from the Swedish National Board of Health and Welfare regarding the treatment of depression in adults [10], antidepressant medication and psychological treatments, including cognitive behavioural therapy, are recommended first-hand, and physical activity is included as an alternative.

In Sweden, approximately 25% of older adults with depressive symptoms receive antidepressant medication, about 3% receive psychological treatment and a majority receive no treatment at all [11, 12]. Psychological treatments are effective for the treatment of depression in adults in general, with better long-term effects than pharmacological treatment [13]. Internationally, studies of various forms of physical activity for older adults have yielded mixed results on mental health outcomes [14–16]. An umbrella review has concluded that physical activity is effective in reducing depressive symptoms in older adults [17]. In a meta-analysis examining the effects of psychological treatments in different age groups, no significant differences were found between adults in general and adults aged 65 years or older [18]. Another meta-analysis has shown that cognitive behavioural therapy for depression is as efficacious for older adults as for young and middle-aged adults [19]. Also, most older adults with depression prefer non-pharmacological treatment to pharmacological treatment for depression [20].

One established psychological treatment for depression is behavioural activation (BA), a brief and structured intervention focusing on increasing engagement in adaptive activities and decreasing engagement in activities that maintain or increase the risk for depression [21]. Central interventions in BA are activity monitoring, which aims to give insight into how activities affect mood, and activity scheduling, which aims to increase the performance of meaningful and enjoyable activities through structured scheduling [22].

Behavioural activation is an extensively evaluated, effective, and relatively simple treatment for depression that can be delivered by health care professionals without extensive training in psychological treatment [23]. Several meta-analyses [23–25] have shown that BA is more effective than control conditions in treating depression. Fewer studies have examined the effect of BA in older adults and many of the existing studies are small [23]. However, a meta-analysis investigating the effect of BA for the treatment of depression in older adults indicate that BA is an effective treatment in this population [21].

One way to facilitate access to healthcare for older adults is to utilize telemedicine [26], that is, providing remote health care through electronic means of communication [27]. A recent meta-analysis investigating telemedicine interventions for depression and anxiety in older adults showed that interventions delivered by internet or telephone are feasible and efficacious [28]. Behavioural activation is feasible and effective for the treatment of depression delivered in a variety of formats including via internet [29], videoconferencing [30–32], and telephone [33–35]. However, it is important to note that studies investigating BA via telephone are small and several lack control group. Few studies have investigated telephone-based BA for older adults. One study by Marti and colleagues found that telephone-based BA reduced depressive symptoms and disability [36]. In a recent study by Gilbody and colleagues, telephone-based BA was found to have positive effects on depressive symptoms and emotional loneliness in older adults with long term conditions during the COVID-19 pandemic [37]. Our research-group conducted a pilot trial of brief telephone-based BA for older adults with depressive symptoms during the COVID-19 pandemic. The results suggest that the treatment is feasible, acceptable, and potentially efficacious [38].

There is a need for fully powered studies of brief behavioural activation for older adults with depression delivered by telephone in a primary care context during non-pandemic conditions as well as studies of behavioural activation in older adults in a Swedish primary care context. Drawing on the promising results from our pilot trial, we will now conduct a study to investigate the telephone-delivered BA treatment for older adults with depression in primary care.

## Objectives and hypothesis

The aim of the current study is to evaluate the effects of a brief telephone-based BA treatment for the treatment of depression in individuals 65 years and older in primary care.

We hypothesise that participants randomised to the treatment condition receiving the BA treatment will show a stronger decrease in depressive symptoms (primary outcome) and improve more on secondary outcomes, compared to participants randomised to the control group receiving treatment as usual (TAU).

## Methods

### Study setting and design

This study is designed as an open label randomised controlled 2-armed parallel group multicentre trial. The study will be conducted in primary care in the Swedish regions of Västmanland, Sörmland, and Uppsala. Initially, four primary healthcare centres (PHCs) will be included in the study, two in Västmanland, one in Sörmland, and one in Uppsala. All PHCs in these three regions received an invitation to participate in the study via the primary healthcare board in each region. Individual PHCs were selected based on logistical factors and their interest in participating. Additional PHCs, within these three regions, may be included if the recruitment of participants is insufficient from the initial four PHCs. A representative from The Swedish Partnership for Mental Health (NSPH) has been involved in the design of the study to improve feasibility for the participants. This RCT protocol is reported according to the recommendations specified in Standard Protocol Items: Recommendations for Interventional Trials (SPIRIT) [39], and the completed checklist is attached in supplementary material 1.

### Participants, recruitment, and randomisation

General practitioners (GPs) at each PHC will be responsible for identifying potential participants and provide interested patients with oral and written information about the trial. Eligible participants should be 65 years or older, present with depressive symptoms, understand Swedish well enough to understand the treatment materials, have access to telephone, and be interested in participating in the trial. Before enrolment, a more thorough clinical assessment is conducted by a study psychologist or study physician and the participants will be given oral information about the study and have opportunity to ask questions before signing an informed consent form. To be enrolled, participants should fulfil criteria for current minor or major depressive disorder according to the Diagnostic and Statistical Manual of Mental Disorders 5th edition, DSM-5 [40]. The exclusion criteria are (1) current psychological treatment, (2) severe depression, (3) elevated suicide risk, (4) severely impaired vision or hearing, (5) current substance use disorder, (6) current or previous manic or hypomanic episodes, (7) current or previous psychotic disorder, and (8) current diagnosis of minor or major neurocognitive disorder or suspected neurocognitive disorder (Mini Mental State Examination result < 25). Concomitant anti-depressant medication is permitted if no changes were made regarding dose or type of anti-depressant medication within the past 8 weeks.

All participants that provide informed consent and fulfil the inclusion criteria and not any exclusion criteria will consecutively be randomised to either the control group receiving TAU or the treatment group receiving TAU with the addition of the BA treatment. The randomisation will be conducted by a study psychologist or study physician after the baseline assessment is completed. Study randomisation will be conducted using Study Randomizer [41], a web-based randomisation service. Participants will be randomly allocated to either control or intervention group (1:1) via random sized blocks of two, four, or six. The researcher conducting the follow-up diagnostic interviews will be blinded to participant allocation, GPs at the PHCs will not be blinded to patient allocation.

## Interventions

### Treatment group

The treatment group will receive TAU with the addition of a five-session telephone-based BA-intervention over an 8-week period. The telephone-based BA-intervention is based on a treatment protocol called Behavioural Activation for Primary Care, BA-PC [42, 43], that was translated and adapted to Swedish by our research group and subsequently used in a pilot study of BA in isolated older adults with depression during the COVID-19 pandemic [38, 44]. Fourteen of the participants were interviewed on their experiences of the treatment [45], and one suggestion from several of the participants was to add one follow-up call, a so-called booster session. Another suggestion that has been implemented in this version of the treatment is to conduct the first treatment session in-person.

In the planned study, the treatment will consist of five treatment sessions, with sessions 1 through 4 conducted once a week and session 5 conducted 4 weeks after session 4. Session 1 will be conducted in-person, and the following sessions will be conducted by telephone. The estimated session length is 45–60 min for session 1 and 30–45 min for sessions 2 through 5. The session outline is described in Table 1. There will be no special criteria for discontinuing or modifying allocated interventions.

**Table 1:** Session outline for the BA treatment

Session no	Session components
1	Present the treatment materials Assess mood and suicide risk Provide psychoeducation about depression Provide treatment rationale for behavioural activation (BA) Provide rationale and instructions for activity log If highly motivated for change, plan activity for coming week
2	Follow-up on mood and suicide risk Review activity log Discuss life goals and values Plan activities aligned with life goals and values for coming week
3	Follow-up on mood and suicide risk Review activity log Troubleshoot any problems carrying out activities Plan activities aligned with life goals and values for coming week
4	Follow-up on mood and suicide risk Review activity log Troubleshoot any problems carrying out activities Plan activities aligned with life goals and values for coming week Instruct the participant to independently plan activities aligned with life goals and values until the booster session
5	Follow-up on mood and suicide risk Review activity log Troubleshoot any problems planning or carrying out activities Review treatment Create a maintenance plan

All study therapists will receive a 1-day training on the BA protocol, provided by a licenced clinical psychologist who also was responsible for the translation of the study manual. The training will include a review of the treatment protocol and the treatment materials, roleplay of critical procedures in the treatment, and discussions about common challenges in BA treatment and ways to handle these challenges. Throughout the study period, all study therapists will attend biweekly online supervision sessions led by the same psychologist that provided the BA training. To further promote adherence to the BA protocol, a checklist of key session components will be filled out by the study therapist at the end of each session.

### Control group

Participants randomised to the control group will receive TAU in accordance with national and regional guidelines which includes antidepressant medication and psychological treatments in first-hand and physical activity as an alternative [10]. The psychological treatments recommended is cognitive behavioural therapy and interpersonal therapy in first-hand and short-term psychodynamic therapy as an alternative. Treatment could vary between general practitioners and PHCs as the guidelines only provide recommendations.

## Outcome measures

### Baseline

**Fig. 1:**
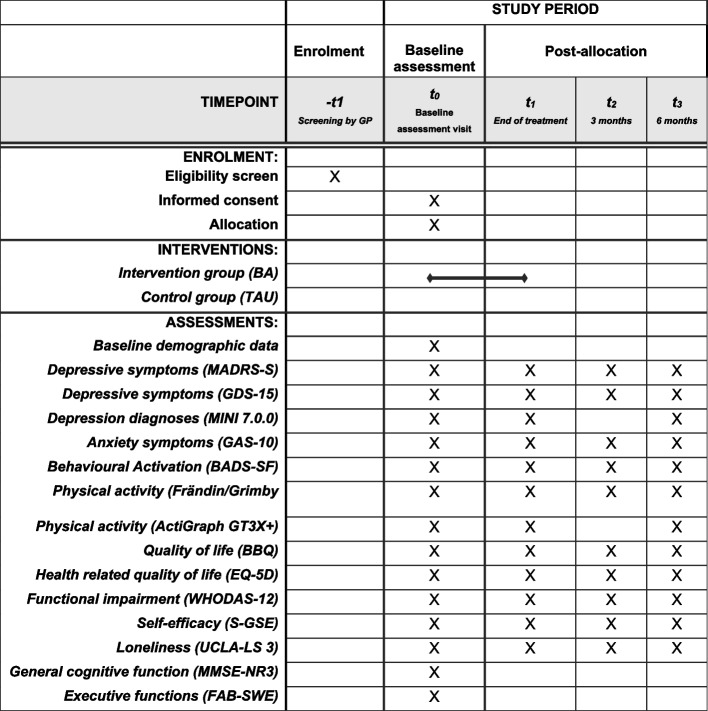
SPIRIT flow diagram: schedule of enrolment, interventions, and assessments

Data will be collected four times, starting with the baseline assessment (Fig. 1). The baseline assessment will include cognitive testing, self-administrated questionnaires, and a structured psychiatric interview. All assessors (study psychologist or study physician) will receive a 1-day training on the assessment procedures and instruments. The battery of questionnaires was tested in a focus group consisting of four individuals ≥ 65 years. Their feedback was used to further enhance the feasibility and layout of the questionnaires without violating the instruments original design.

Global cognitive status will be measured using the Mini Mental State Examination [46] – Swedish revision, MMSE-NR3, a 30-point cognitive screening test. Executive functioning will be measured with the Frontal Assessment Battery – Swedish version, FAB-SWE [47], a 6-item screening test with scores ranging from 0 to 18.

### Primary outcome

The primary outcome is depressive symptoms measured with the Montgomery-Åsberg Depression Rating Self-rating Scale, MADRS-S [48]. The MADRS-S is a nine-item questionnaire designed to measure depression severity during the past 3 days.

### Secondary outcomes

The secondary outcomes include depression diagnosis, activity level, and self-rated anxiety, daily functioning, quality of life, self-efficacy, and loneliness.

The Mini International Neuropsychiatric Interview (MINI 7.0.0) [49] is a structured psychiatric interview that will be used to assess psychiatric diagnoses, including depression diagnosis, and comorbidity at baseline. The depressive episode module in MINI 7.0.0 will be conducted by telephone to assess depression diagnosis at post-treatment and 6-month follow-up. The section for antisocial personality disorder will be excluded due to irrelevance to the current study. Depressive symptoms will be measured using the Geriatric Depression Rating Scale 15-item short form (GDS-15) [50]. This questionnaire is used to identify depression in older individuals with scores ranging from 0 to 15, with higher scores indicating more depressive symptoms Anxiety symptoms will be measured with the Geriatric Anxiety Scale – 10 item version, GAS-10 [51], with a score ranging from 0 to 30. The respondents rate their anxiety symptoms on a 4-point scale, with higher scores indicating higher levels of anxiety.

Self-rated avoidance and activation will be measured using the Behavioural Activation for Depression Scale—Short Form (BADS-SF) [52], a 9-item scale with scores ranging from 0 to 54, with higher scores indicating a higher degree of activation and lower degree of avoidance. This instrument was developed specifically to measure activation level during BA treatments.

Subjective feelings of loneliness and social isolation will be measured using the UCLA Loneliness Scale version 3 (UCLA-LS 3) [53], a 20-item scale with scores ranging from 20 to 80 points. Higher scores indicate higher degrees of loneliness. There is data supporting the reliability and validity of using the UCLA-LS 3 in assessing loneliness in a variety of populations, including older adults [53].

Physical activity level will be measured by using the Frändin/Grimby activity scale, in which people estimate their activity on a 6-point scale [54]. The scale has been shown to be valid for assessing physical activity among older adults [54, 55]. The participants will also be instructed to wear the ActiGraph GT3X + accelerometer on an elastic belt around their waist for seven consecutive days, all waking hours (except when bathing or showering). The ActiGraph GT3X + accelerometer measures movement (acceleration) in up to 3 orthogonal axes via electrical impulses. The ActiGraph (Pensacola, Florida) accelerometers are widely used in research because they have shown high validity and reliability [56, 57]. The accelerometer will be used to objectively define the participants levels of physical activity at baseline, post-treatment, and 6-month follow-up.

The New General Self-Efficacy Scale (S-GSE) is an 8-item measure with a 4-point scale that assesses self-efficacy, that is how much people believe they can achieve their goals, despite difficulties [58]. Higher scores indicate a higher degree of self-efficacy.

Functional impairment will be assessed with the World Health Organization (WHO) Disability Assessment Schedule 12-item (WHODAS-12), a self-rating scale with 12 items [59]. Higher scores indicate greater functional impairment.

The Brunnsviken Brief Quality of life scale (BBQ) measures importance-adjusted satisfaction and has been recommended for use in psychological and psychiatric research and practice [60]. The BBQ is a self-administered questionnaire using 12 questions to address six areas: Leisure time, View on life, Creativity, Learning, Friends and Friendship, and View of self. Health-related quality of life and estimation of quality-adjusted life years (QALY) for health economic evaluations will be measured using the EuroQol-5 Dimensions-5 Level Scale (EQ-5D) [61], a 5-item scale measuring health status with regards to mobility, self-care, usual activities, pain/discomfort, and anxiety/depression.

## Sample size

**Fig. 2:**
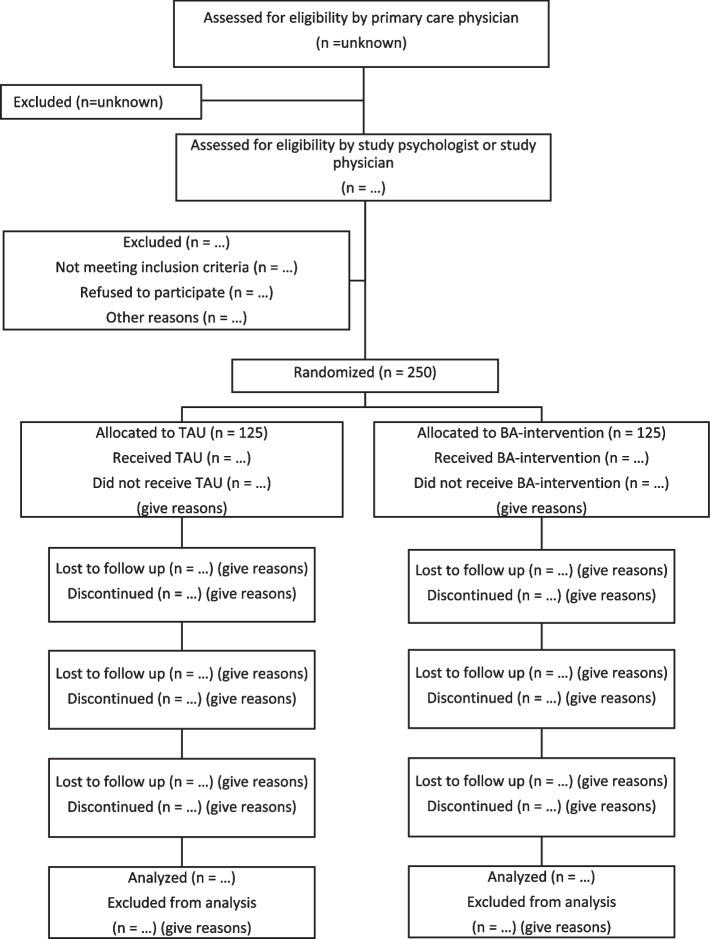
Modified CONSORT flow diagram for individual randomised, controlled trials of nonpharmacologic treatment

A total of 250 participants will be included in the trial. Sample size calculation is based on a previous meta-analysis of Behavioural Activation in older adults with mean effect sizes of 0.72 at post-treatment and 0.44 at follow-ups at 3–6 months [21], which is in line with our pilot study of telephone-based BA for older adults yielding an effect size of 0.85 at post-treatment [38] and 0.41 at 6 months post-treatment [44]. An effect size of 0.4 was chosen, based on previously mentioned meta-analysis and pilot study but also following recommendations on the lower value for an effect size corresponding to a medium-sized effect in gerontological research [62]. According to G*Power, 250 participants are needed in total to achieve 80% power with a significance level of 0.05 and a drop-out rate of 20% for analysis of co-variance, ANCOVA. The flow of this study will follow the Modified CONSORT Statement extension for individual randomised controlled trials of non-pharmacological treatments (Fig. 2) [63].

## Retention

A text message reminder will be sent to the potential participant 1 day before the clinical assessment. The participants will receive a timeline illustrating the different components of their participation in the study; an updated version of the timeline with the addition of an instruction letter for the assessments will be provided at each follow-up. The participants will be reminded of the questionnaires and the accelerometer during the telephone interview at post-treatment and 6-month follow-up.

Each healthcare centre will have a contact person representing the study. The contact person will visit the healthcare centre regularly to answer questions and remind the GPs about the trial. Posters will be put up in staff areas as a reminder and GPs will receive notepads listing the required participant information and complete inclusion/exclusion criteria to facilitate recruitment of eligible participants.

## Data management

All data forms and data files will be pseudonymised, and the code key will be stored separate. Access to the code key is restricted to researchers in the research group and study therapists taking part in initial assessment and treatment. All data will be managed using Vesta, a secure storage for research data provided by Uppsala university. If a participant chooses to discontinue their participations in the study, all the information up until then is used in the analyses but no additional information is collected.

The PI and study coordinator are certified in Good Clinical Practice (GCP), and the enrolled Ph.D. students has also been trained in GCP. General Data Protection Regulation (GDPR) and necessary agreements will be ensured according to the routines at the Centre for Clinical Research in Region Västmanland and Uppsala university. The research group, consisting of the PI, Ph.D. students, and their supervisors, will attend monthly meetings to assure that the data collection is conducted in accordance with the study protocol. Additional monitoring of trial progress is not deemed necessary due to a low risk of harm [38].

## Statistical methods

The difference between the intervention group and control group in depressive symptoms post-intervention measured with MADRS-S will be estimated using analysis of covariance (ANCOVA) with baseline values on the MADRS-S as covariate as well as with the between-group effect size post-intervention using Cohen’s *d*. Difference from baseline between the groups post-intervention and long-term follow-ups (3 and 6 months) will be analysed separately with repeated measures ANOVA. All continuous secondary outcome measures will be estimated using ANCOVA and repeated measures ANOVA, whereas discrete outcomes (i.e. depression diagnosis) will be estimated using logistic regression. Attrition will be analysed using *T*-test for continuous measures (age, depression severity on the MADRS-S, cognitive functioning on the MMSE and FAB-Swe) and chi-square for sex. Analyses will be conducted both using intention-to-treat (ITT) as a primary analysis and per-protocol as a secondary analysis. In the ITT, all randomised participants will be included, whereas only participants that adhered to the treatment or control-condition will be included in the per-protocol analyses. Missing data will be handled by using multiple imputation. Stopping guidelines for trial termination due to negative effects is not applicable and no interim analyses are planned.

## Harms

No serious adverse events (SAEs) were reported in the pilot trial [38]. There is no anticipated harm and compensation for trial participation. The study therapists will continuously evaluate the participants' condition and suicide risk at each treatment session in the treatment group. We will adhere to the local guidelines for elevated suicide risk in primary care. Serious adverse events will be reported to the research team immediately after obtaining knowledge about them. Any harms reported to the research team will be described in future trial publications.

## Discussion

Depressive symptoms among older adults are common [2–4] but are often unrecognised and untreated [11]. Few older adults receive psychological treatment [12] even though it is often preferred over pharmacological treatment [20] and is recommended as a first-hand treatment in the guidelines from the Swedish National Board of Health and Welfare [10].

The aim of this trail is to investigate the effect of a brief telephone-based BA treatment for depression in older adults in primary care. Although BA is an established and extensively evaluated treatment for depression [23], there is a need for studies evaluating the effect of brief behavioural activation for older adults with depression delivered by telephone in a primary care context and studies of Behavioural activation in older adults in a Swedish primary care context.

Although efforts have been made in the planning of the study, it still has some limitations that will be addressed. First, neither the participants nor the GPs at the PHCs will be blinded to participant allocation. Hence, expectations may influence the results, and the content of TAU given by the GPs may be affected. To reduce the risk of bias in the follow-up diagnostic interviews, they will be conducted by a researcher blinded to participant allocation. Second, we are aware that the recruitment of participants could be more challenging than initially anticipated. To facilitate recruitment and collaboration with the individual PHCs, the logistical routines have been designed in cooperation with the PHCs, and each PHC will have an appointed contact person responsible for visiting the PHC regularly and answering any questions. The GPs will be provided with materials intended to make their recruitment of participants easier. We also have the possibility to include additional PHCs if needed.

If the telephone-based BA treatment proves to be effective, it can, with its brief and simple format, have the potential to improve first-line treatment of depression in older adults in primary care, consequently reducing morbidity and mortality within this growing population. The simple format facilitates implementation in the healthcare system since the treatment can be delivered by a variety of health care professionals, without the need for comprehensive training in psychological treatment. Increasing the availability and accessibility to effective psychological treatment for depression in older adults is needed to meet future demographic changes.

## Trial status

Protocol version 1, 2024–02-28. Recruitment began March 5, 2024, and preliminary date for recruitment completion is June 30, 2027. If there will be any important protocol modification, this will render an additional application to the Swedish ERA and a modification of the trial registration.

## Supplementary Information

Supplementary Material 1. SPIRIT Checklist for *Trials*.

## Data Availability

Data are available upon reasonable request. Qualified researchers may request access to patient-level data and related study documents, including the study protocol with any amendments, and dataset specifications. Patient-level data will be anonymised, and study documents will be redacted to protect the privacy of trial participants. Results from the trial will be communicated through future trial publications.

## Abbreviations

BA: Behavioural activation
BADS-SF: Behavioural Activation for Depression Scale—Short Form
BA-PC: Behavioural Activation for Primary Care
BBQ: Brunnsviken Brief Quality of life scale
DSM-5: Diagnostic and Statistical Manual of Mental Disorders 5th edition
EQ-5D: EuroQol-5 Dimensions-5 Level Scale
ERA: Ethical Review Authority
FAB-SWE: Frontal Assessment Battery – Swedish version
GAS-10: Geriatric Anxiety Scale – 10 item version
GDS-15: Geriatric Depression Rating Scale 15-item short form
GP: General practitioners
ITT: Intention-to-treat
MADRS-S: Montgomery-Åsberg Depression Rating Self-rating Scale
MINI 7.0.0: Mini International Neuropsychiatric Interview version 7.0.0
MMSE-NR3: Mini Mental State Examination – Swedish revision
NSPH: The Swedish Partnership for Mental Health
PHCs: Primary Healthcare Centres
SAEs: Serious adverse events
S-GSE: New General Self-Efficacy Scale
SPIRIT: Standard Protocol Items: Recommendations for Interventional Trials
TAU: Treatment as usual
UCLA-LS 3: UCLA Loneliness Scale version 3
WHO: World Health Organization
WHODAS-12: WHO Disability Assessment Schedule 12-item
